# Comment on “Designing Robust N-of-1 Studies for Precision Medicine: Simulation Study and Design Recommendations”

**DOI:** 10.2196/16179

**Published:** 2020-09-15

**Authors:** Reid D Landes

**Affiliations:** 1 Department of Biostatistics University of Arkansas for Medical Sciences Little Rock, AR United States

**Keywords:** sample size, misleading statements

In “Designing robust N-of-1 studies for precision medicine: Simulation study and design recommendations” by Percha et al [1], the authors use misleading language when speaking about the required numbers of samples regarding results in Figure 4a. For example, they write on page 8:

In Figure 4a, we see that for effect sizes of 0.1, 0.2, and 0.3, more than 100 samples are needed to obtain a power of 0.8 (at a standard 5% significance level). For an effect size of 0.4, at least 100 samples are needed. For effect sizes of 0.5, 0.6, 0.7, 0.8, 0.9, and 1.0, the numbers of samples needed to attain a power of 0.8 are approximately 65, 45, 35, 26, 21, and 18, respectively. [Figure 4]

Since Figure 4a is exactly equivalent to power curves from a two-sample, equal-variance *t* test (see Figure 1; generating R code provided in Textbox 1), the numbers of samples are for *one* of the two treatments; thus, the total numbers of samples are doubled. An easy fix in most instances of the unclear language is to add “per treatment” after “samples.” I provide a list of potential clarifying edits to the article’s text below (but may have missed some instances):

- Figure 4c caption: “(ie, number of samples *per treatment*, with sampling rate fixed at 1 sample per time unit)”
- Figure 4a and 4b: the label for the horizontal axis should be “Number of samples *per treatment*”
- Page 8: “In Figure 4a, we see that for effect sizes of 0.1, 0.2, and 0.3, more than 100 samples *per treatment* are needed to obtain a power of 0.8 (at a standard 5% significance level). For an effect size of 0.4, at least 100 samples *per treatment* are needed. For effect sizes of 0.5, 0.6, 0.7, 0.8, 0.9, and 1.0, the numbers of samples *per treatment* needed to attain a power of 0.8 are approximately 65, 45, 35, 26, 21, and 18, respectively.”
- Page 9: “For an effect size of 0.5 and σ_p_=0.0, 0.4, 0.8, 1.2, 1.6, 2.0, the numbers of samples *per treatment* needed to obtain a power of 0.8 are 61, 76, 89, 111, 135, and 176, respectively. For an effect size of 1.0, the numbers of samples *per treatment* needed are 20, 24, 28, 34, 43, and 53, respectively.”
- Page 9: “For σ_p_=0.0, 0.4, 0.8, 1.2, 1.6, 2.0 and α=0.1, the numbers of samples *per treatment* required are 36, 64, 110, 174, 228, and 250, respectively. For α=10.0, the numbers of samples *per treatment* required are only 20, 23, 28, 34, 42, and 53, respectively.”

**Figure 1:**
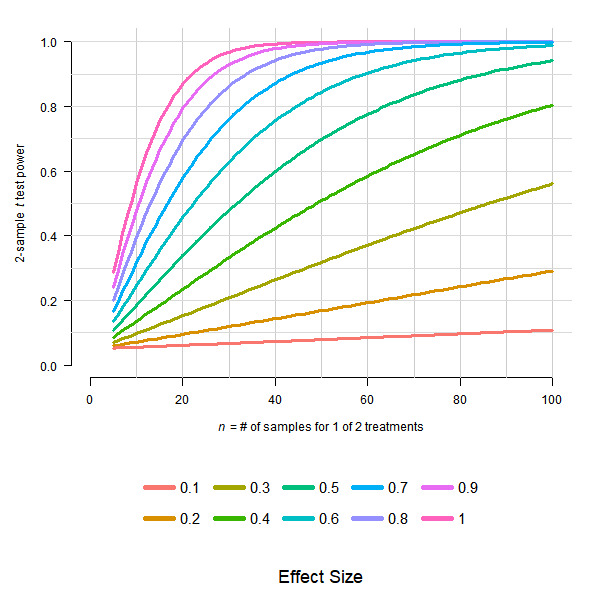
For effect sizes ranging from 0.1 to 1.0, power of a 0.05 level two-sample *t* test plotted by *n*, the number of samples in one treatment group. Total sample size is assumed to be *2n*.

**R code.**

#--- R code for generating Figure 1.

library(pwr)

color <- c("#F8766D", "#D89000", "#A3A500", "#39B600", "#00BF7D", "#00BFC4",

           "#00B0F6", "#9590FF", "#E76BF3", "#FF62BC")

 

#--- Producing the power plot (upper portion of figure)

plot(50, 1, type = 'n', xlim = c(0,100), ylim = c(0,1), axes = FALSE,

     ylab = substitute(paste("2-sample ", italic('t'), " test power")),

     xlab = substitute(paste(italic('n'), " = # of samples for 1 of 2 treatments") ) )

axis(1, at = seq(0, 100, 20)); axis(2, at = seq(0, 1, .2), las = 2)

abline(v = seq(10, 100, 10), col = 'gray80')

abline(h = seq(.1, 1, .1), col = 'gray85')

 

#--- Filling in the power plot

for(.d in 10:1/10){

   tmp.power <- NULL

   for( .n in 5:100){

     #--- Computes power for a 2-sample t-test, each sample with n observations.

     p <- pwr.t.test(n = .n, d = .d, sig.level = .05, power = NULL,

                     type = "two.sample", alternative = "two.sided")$power

     tmp.power <- c(tmp.power, p)

   }

   lines(5:100, tmp.power, lwd=3, col=color[.d*10])

}

 

#--- Producing the the legend (lower portion of figure)

plot(50, 1, type = 'n', xlim = c(0,100), ylim = c(.25, .75),

      axes = FALSE, xlab = 'Effect Size', ylab = '', cex.lab=1.5)

x <- 15

for(iter in seq(2,10,2)){

   mult <- (iter/2)

   .x <- x*mult

   segments( .x-3, 0.67, .x+3, lwd=5, col= color[iter-1])

   text(.x+3, .67, pos=4, (iter-1)/10, cex=1.25)

   segments( .x-3, 0.33, .x+3, lwd=5, col= color[iter] )

   text(.x+3, .33, pos=4, (iter)/10, cex=1.25)

}

## References

[1] Percha Bethany, Baskerville Edward B, Johnson Matthew, Dudley Joel T, Zimmerman Noah (2019). Designing Robust N-of-1 Studies for Precision Medicine: Simulation Study and Design Recommendations. J Med Internet Res.

